# Triglycerides, independent of Ferriman Gallwey Score, is a main determinant of free testosterone index in PCOS

**DOI:** 10.12688/f1000research.16815.1

**Published:** 2019-01-23

**Authors:** Andon Hestiantoro, Putri Deva Karimah, Amalia Shadrina, Budi Wiweko, R. Muharam, Brilliant Putri Kusuma Astuti

**Affiliations:** 1 Reproductive Immunoendocrinology Division, Department of Obstetrics and Gynecology, Faculty of Medicine Universitas Indonesia, Dr. Cipto Mangunkusumo General Hospital, Jakarta Pusat, DKI Jakarta, 10430, Indonesia; 2 Indonesian Reproductive Medicine Research and Training Center (Inarepromed, Faculty of Medicine Universitas Indonesia - (Indonesian Medical Education and Research Institute (IMERI), Dr. Cipto Mangunkusumo General Hospital, Jakarta Pusat, DKI Jakarta, 10430, Indonesia; 3 Department of Obstetrics and Gynecology, Faculty of Medicine Universitas Indonesia, Dr. Cipto Mangunkusumo General Hospital, Jakarta Pusat, DKI Jakarta, 10430, Indonesia

**Keywords:** Polycystic Ovarian Syndrome, Hyperandrogenism, Free Testosterone Index, Hirsutism, Ferriman Gallwey Score, Triglyceride

## Abstract

**Background:** Polycystic Ovarian Syndrome (PCOS) is the most common endocrinopathy in women of reproductive age, affecting 5-20% of women worldwide. Hyperandrogenism, as the primary characteristic of PCOS, is not always present in every patient. The hyperandrogenic phenotype of PCOS patients is influenced by both hormonal and metabolic dysfunctions. Therefore, this study aims to determine the correlation between hormone profile, lipid profile, and clinical profile with free testosterone index in subjects with PCOS.

**Methods:** This prospective cross-sectional study was conducted in the Dr. Cipto Mangunkusumo General Hospital between July 2014 and December 2016. The study involved 76 women with PCOS, who were classified into 2 subgroups: 39 subjects in the hyperandrogenism group and 37 subjects in the non-hyperandrogenism group. Each subject underwent physical examination, blood sample collection, and USG examination. Bivariate analysis was done using independent t-tests and Mann Whitney U-tests, while multivariate analysis was done using logistic regression.

**Results:** Triglyceride and testosterone level showed weak (r = 0.232, p = 0.044) and moderate (r = 0.460, p ¡ 0.001) positive correlation with FTI, while SHBG level showed moderate negative correlation (r = -0.483, p ¡ 0.001). Triglyceride was also found to be determinant of hyperandrogenism condition in PCOS patient (OR 0.02, 95% CI 0.00–0.04, p = 0.013). However, there was no significant difference observed between FGS and hyperandrogenism (p = 0.43).

**Conclusions:** Triglycerides, testosterone, and SHBG were associated with hyperandrogenism in PCOS patients, while FGS showed no such association.

## Introduction

Polycystic Ovarian Syndrome (PCOS) is the most common endocrinopathy in women of reproductive age, affecting 5–20% of women, depending on the diagnosis criteria used ^1^. PCOS is a complex disorder with a wide range of physical manifestation which is primarily characterized by hyperandrogenism or hyperandrogenemia, chronic anovulatory cycle, and polycycstic ovarian morphology. PCOS patients are considered to be at increased risk of developing several co-morbidities, such as diabetes, dyslipidemia, dysmetabolic syndrome, hypertension, obesity, obstructive sleep apnea, mental health disorders, and increased risk of cardiovascular diseases ^2– 4^.

Hyperandrogenism is one of the hallmark pathophysiological features of PCOS. Along with insulin resistance, hyperandrogenism causes metabolic derangements and some cutaneous symptoms, such as hirsutism, acne, and androgenic alopecia. The extent of hyperandrogenic clinical presentation varies among individuals and is affected by several factors, such as genetic polymorphism, inappropriate epigenetic reprogramming, metabolic factors, and other environmental factors ^3, 5^. An intricate interrelationship between environmental factors and aberrant micro-RNA expression has been proposed as the epigenetic mechanism underlying the development of PCOS ^5^. It can be implicitly recognized that the phenotypic heterogeneity may illustrate the differences in their underlying genetic and metabolic pathophysiology, including the abnormalities of insulin regulation and lipid metabolism.

Besides its prominent aspect in the pathophysiology of PCOS, hyperandrogenism also possesses a fundamental role in achieving a PCOS diagnosis. Hyperandrogenism, as one of the three diagnostic criteria of PCOS, can be identified with physical examination and laboratory evaluation. Hirsutism is the most frequently found clinical manifestation of hyperandrogenism, accounting for approximately 70–80% of all PCOS cases ^3, 6, 7^. The modified Ferriman-Gallwey Score (mFGS) is used to measure the degree of terminal hair growth in several body sites and total score of *≥* 8 is considered hirsutism ^8^. Biochemical assessment of hyperandrogenism involves the evaluation of several parameters, such as free testosterone index (FTI), free androgen index (FAI) or dehydroepiandosterone sulfate. Elevated levels of one of those markers indicate the presence of hyperandrogenemia ^9^. However, elevated androgen levels are not always accompanied by obvious peripheral manifestations. Many studies found that only half of women with hirsutism have elevated levels of androgen hormones and only one-third of women with elevated androgen hormones have hirsutism ^7^. Hirsutism is a multifactorial condition and androgen only plays a partial role in its occurrence. These findings suggest that hirsutism might not be the most suitable marker for identifying the elevation of androgen levels in PCOS patients.

It is important to evaluate further which factors correlate with testosterone levels reflected with free testosterone index in subject PCOS. We hypothesized that the hyperandrogenic phenotype of PCOS patients is influenced by both hormonal and metabolic dysfunctions. Therefore, this study aims to determine the association between hormone profile, lipid profile, and clinical profile with free testosterone index in subjects with PCOS.

## Methods

### Study background

This was a cross-sectional study conducted at Dr. Cipto Mangunkusumo General Hospital, Jakarta from July 2014 until December 2016. Sample size was determined using Lemeshow sample size formula as presented below:

N = (Z *α* ^2^ x p(1-p)/d ^2^

N represents the minimum sample size. Z *α* represents standard normal deviation corresponding to 100% *α* (1.96) p represents the proportion of PCOS patients in Cipto Mangunkusumo General Hospital (45.7 %) d represents precision (12 %) According to that formulation, the minimum sample size of this study was 66.2 subjects. To account for potential post-enrollment drop out, additional 10% subjects were assigned to the study, resulting in a minimum sample size of 72 subjects. Subjects were recruited in-person consecutively from patients who came to the gynecology clinic with the chief complaint of irregular menstrual cycle. The recruitment of subjects was terminated once the minimum sample size has been achieved. Data and sample collection were performed immediately after patients agreed to participate in this study and were conducted during patient’s visit to gynecology clinic. All subjects were recruited based on the inclusion criteria, which included women between the age of 18 and 40 years old, who had been diagnosed with PCOS based on Rotterdam consensus criteria, have not consumed any PCOS or hormonal medication in the past 3 months, and were willing to participate in this study. Women who were pregnant or currently breastfeeding; those with history of uterine and other adnexal abnormalities, disorder of adrenal gland function, primary hypothalamic – hypophyseal disorder, ovarian tumor, disorder of prolactin secretion, unexplained abnormal uterine bleeding, and with previous history of thromboembolic or cerebrovascular disorders were excluded from this study.

### Ethics

The study protocol was approved by Ethics Committee of Faculty of Medicine Universitas Indonesia and Dr. Cipto Mangunkusumo Hospital, with reference number of 818/UN2.F1/ETIK/X/2016. Prior to the beginning of this study, subjects were informed about the protocol of the study and were asked to sign written consent form.

### Baseline measurements

Those who met the aforementioned criteria then underwent medical history taking and physical examination, including waist circumference, body weight, and body height measurements; hirsutism index through FGS ^10^; and gynecologic examination.

FGS is a visual instrument which is widely used to evaluate the excess growth of terminal hair in several body areas. There are 9 androgen sensitive areas which are assessed in FGS, and each area is assigned with value from 1 to 4 according to the thickness of the hair growth. These areas are lip, chin, chest, upper abdomen, lower abdomen, upper arm, thigh, upper back, and lower back. The cut off point at which hirsutism diagnosis is made varies depending on race and ethnicity. However, in general a total score equal to or more than 8 signifies hirsutism.

Ultrasonography was done to assess polycystic features. Blood samples were collected during the initial study for fasting plasma glucose, 2-hour postprandial plasma glucose, insulin, Homeostatic Model Assessment Insulin Resistance (Homa-IR), prolactin, LDL, HDL, Triglyceride, SHBG, TSH, LH, FSH, FTI, and testosterone examination. Subjects were instructed to avoid eating or drinking anything for 9–12 hours before the blood sample is collected. Approximately 10 mL of venous blood samples were drawn from each subject and were collected in several Vacutainers® blood collection tubes. After initial blood collection, subjects were instructed to have a full-course meal or a meal with at least 75 g of carbohydrates. Two hours following the meal, venous blood samples were drawn again to evaluate the 2 hour postprandial plasma glucose. All specimens were stored in a *−*70ª freezer before being transported to the lab for further analysis. Biochemical analysis was conducted at Prodia clinical laboratory. Fasting and 2-hour postprandial plasma glucose were determined using ARCHITECT Glucose Reagent Kit (Abbott Diagnostics, Illinois, USA). Plasma insulin was determined using ARCHITECT Insulin Reagent Kit (Abbott Diagnostics, Illinois, USA). HOMA-IR was calculated according to this formula = fasting insulin (in *µ*IU/ml) X fasting plasma glucose (in mg/dL). Prolactin level was determined using ADVIA Centaur Prolactin Kit (Siemens Healthineers Global, New York, USA). LDL-C level was determined using Sekisui Cholesterol LDL Kit (Siemens Healthineers Global, New York, USA). HDL-C level was determined using Sekisui Cholesterol HDL Kit (Siemens Healthineers Global, New York, USA). Triglyceride level was determined using ADVIA Chemistry Triglyceride Reagent Kit (Siemens Healthineers Global, New York, USA). SHBG level was determined using ADVIA Centaur Immunoassay Kit (Siemens Healthineers Global, New York, USA). TSH level was determined using ADVIA Chemistry TSHs Reagent Kit (Siemens Healthineers Global, New York, USA). LH level was determined using ADVIA Centaur LH Kit (Siemens Healthineers Global, New York, USA). FSH level was determined using ADVIA Centaur FSH Kit (Siemens Healthineers Global, New York, USA). Testosterone level was determined using Testosterone II Kit (Roche Diagnostics, Risch-Rotkreuz, Switzerland). Free Testosterone Index (FTI) was calculated according to this formula = Total Testosterone (in nmol/L) /SHBG (in nmol/L) x 100.

### Subject classification

The subjects were classified into two groups according to the results of their FTI tests: hyperandrogenism and non-hyperandrogenism groups. The diagnosis of hyperandrogenism was made according to subject’s FTI level. Subjects with FTI measurement equal to or greater than 5 were classified into hyperandrogenism group, while subjects with FTI measurement less than 5 were classified into non-hyperandrogenism group. Comparative analysis was performed between these two groups based on the variables mentioned above. The primary study outcome of this study was to determine the correlation between FGS and FTI, and also factors that contribute to hyperandrogenism phenotype in PCOS patients.

### Statistical analysis

Data obtained from the subjects were recorded in case registration forms and were analyzed using Statistical Package for the Social Sciences (SPSS) version 20. Univariate analysis was done by converting valid data into tables containing mean and median values, as well as their distribution. Bivariate analysis was conducted using independent T-test and Mann-Whitney U-test to compare variables, such as age, body mass index (BMI), body weight, body height, waist circumference, FGS, fasting blood glucose, 2-hour postprandial plasma glucose, insulin, Homa-IR, prolactin, LDL, HDL, Triglyceride, SHBG, TSH, LH, FSH, and testosterone, between hyperandrogenism and non-hyperandrogenism groups. P < 0.05 was considered significant. Among those variables, multivariate analysis using logistic regression was performed on variables with p < 0.25. Finally, correlation analysis was performed using Pearson’s and/or Spearman’s test according to the normality of data distribution. Correlation analysis was performed to quantify the association between the biochemical parameters and free testosterone index in PCOS patients.

## Results

### Baseline characteristics

The 76 subjects participating in this study were classified into two groups: 37 in the PCOS without hyperandrogenism group and 39 in the PCOS with hyperandrogenism group. Most of the subjects were in their mid-20s, overweight (according to Asia Pacific WHO classification), and had central obesity. The subjects’ body weights ranged from 50.5 to 101 kg and the FGS ranged from 1 to 11. Most of the subjects showed good blood glucose profiles: 90.8% subjects had normal fasting blood glucose and 53% subjects had normal 2-hour post-prandial blood glucose. Approximately 94.7% and 82.9% subjects had abnormal LDL and HDL levels, respectively, but most (76.3%) had normal triglyceride levels. The median of insulin level was 13.6 *µ*IU/ml, Homa-IR was 2.89, prolactin level was 9.4 ng/ml, SHBG level was 21.48 nmol/l, TSH level was 1.62 *µ*IU/ml, LH level was 10.7 *µ*IU/ml, FSH level was 6.3 *µ*IU/ml, and testosterone level was 37.55 ng/dl. Details of subject characteristics are shown in Table 1.

**Table 1.** Characteristics of the Study Population

Characteristic	Value (n = 76)
Age	28 (23–35) years old
Body mass index	27.6 (21.5–41) kg/m ^2^
Body weight	0.6 (50.5–101) kg
Waist circumference, cm	92.01 ± 9.79 cm
Ferriman Gallwey score	3 (1–11)
Fasting blood glucose	90 (68 –105) mg/dL
2-hour post prandial Blood glucose	90112.5 (56 – 237) mg/dL
Insulin level	13.6 (8.9 – 36.8) *μ*IU/ml
Homa-IR level	2.89 (2.05 – 8.99)
Prolactin level	9.4 (4.4 – 32) ng/ml
LDL level	128.5 ± 31.5 mg/dL
HDL level	42.51 ± 6.59 mg/dL
Triglyceride level	109.5 (45 – 390) mg/dL
SHBG level	21.48 (7.06 – 65.99) nmol/l
TSH level	1.62 (0.01 – 8.94) *μ*IU/ml
LH level	10.7 ± 4.52 *μ*IU/ml
FSH level	6.3 ± 1.61 *μ*IU/ml
Testosterone level	37.55 (6.94 – 122.6) ng/dL

### Comparison of the two groups

Within bivariate analysis, significant differences between hyperandrogenism and non-hyperandrogenism group were observed in some characteristics, such as triglyceride level (p = 0.01), SHBG level (p = 0.01), and testosterone level (p = 0.04). The hyperandrogenism group had significantly higher level of triglyceride and testosterone, but lower SHBG level. On the other hand, no significant difference was observed between hirsute appearance, which was measured with FGS, and FAI (p = 0.43) (see Table 2).

**Table 2.** Bivariate Analysis.

Characteristic	Non-Hyperandrogenism Group	Hyperandrogenism Group	p value
Age	27 (23 – 34) years old	29 (23 – 35) years old	0.13
Body Mass Index (BMI)	26.89 (21.5 – 41) kg/m ^2^	29 (22.9 – 38.3) kg/m ^2^	0.40
Body Weight	72.5 (50.5 – 101) kg	70.3 (56 – 97.8) kg	0.68
Body Height	1.58 (1.5 – 1.73) m	1.59 (1.45 – 1.68) m	0.91
Waist Circumference	91.83 ± 11.67 cm	92.17 ± 7.76 cm	0.88
Ferriman Gallwey Score (FGS)	2 (1 – 9)	3 (1 – 11)	0.43
Fasting Blood Glucose	90 (70 – 103) mg/dL	89 (68 – 105) mg/dL	0.71
2-Hour Post Prandial Blood Glucose	110 (78 – 237) mg/dL	115 (56 – 208) mg/dL	0.46
Insulin Levels	13.6 (8.9–30.5) *μ*IU/ml	14.7 (9.3 – 36.8) *μ*IU/ml	0.07
Homa-IR Levels	2.75 (2.2 – 7.22)	3.18 (2.05 – 8.99)	0.12
Prolactin Levels	9.3 (4.5 – 26.2) ng/ml	9.8 (4.4 – 32) ng/ml	0.62
LDL Levels	140.62 ± 26.9 mg/dL	46, 09 ± 21, 51 mg/dL	0.23
HDL Levels	42.75 ± 6.49 mg/dL	42.28 ± 6.75 mg/dL	0.75
Triglyceride Levels	95 (45 – 228) mg/dL	122 (69 – 390) mg/dL	0.01
SHBG Levels	23.35 (11.58 – 53.91) nmol/l	19.54 (7.06 – 65.99) nmol/l	0.01
TSH Levels	1.62 (0.01 – 8.94) *μ*IU/ml	1.66 (0.3 – 6.11) *μ*IU/ml	0.15
LH Levels	10.36 ± 5.47 *μ*IU/ml	11.02 ± 3.44 *μ*IU/ml	0.53
FSH Levels	6.27 ± 1.66 *μ*IU/ml	6.47 ± 1.57 *μ*IU/ml	0.59
Testosterone Levels	30.36 (6.94 – 122.6) ng/dL	40.43 (6.94 – 83.37) ng/dL	0.04

Spearman analysis was conducted to evaluate the correlation between these factors and FTI. From the analysis, it was found that triglyceride and testosterone level showed weak (r = 0.232) and moderate (r = 0.460) positive correlations with FTI, while SHBG level showed a moderate negative correlation (r = -0.483) (see Table 3).

**Table 3.** Correlation of Triglyceride, SHBG, Testosterone Level with FTI.

Biochemical Parameter	r	p
Triglyceride level	0.232	0.044
Testosterone level	0.460	< 0.001
SHBG level	- 0.483	< 0.001

Multivariate analysis was conducted using logistic regression, including four variables such as triglyceride level, insulin level, Homa-IR level, and LDL level. Among these variables, triglyceride was found to be an important determinant of hyperandrogenism condition in PCOS patients ( Table 4).

**Table 4.** Multivariate Analysis.

Step	Variables	B	S.E.	Wald	df	Sig.	Exp(B)	95% CI for Exp(B)
1	Insulin	0.274	0.216	1.611	1	0.204	1.315	0.861 - 2.008
	Triglyceride	0.010	0.005	4.800	1	0.028	1.010	1.001 - 1.019
	Homa-IR	-0.952	0.852	1.248	1	0.264	0.386	0.073 - 2.051
	LDL	-0.010	0.008	1.682	1	0.195	0.990	0.974 - 1.005
	Constant	-0.759	1.324	0.328	1	0.567	0.468	
2	Insulin	0.040	0.042	0.917	1	0.338	1.041	0.959 - 1.131
	Triglyceride	0.009	0.004	4.411	1	0.036	1.009	1.001 - 1.018
	LDL	-0.011	0.008	1.896	1	0.169	0.989	0.973 - 1.005
	Constant	-0.248	1.269	0.038	1	0.845	0.780	
3	Triglyceride	0.011	0.004	5.573	1	0.018	1.011	1.002 - 1.019
	LDL	-0.010	0.008	1.685	1	0.194	0.990	0.974 - 1.005
	Constant	0.146	1.188	0.015	1	0.902	1.157	
4	Triglyceride	0.010	0.004	5.360	1	0.021	1.010	1.002 - 1.019
	Constant	-1.219	0.581	4.411	1	0.036	0.295	

Anthropometric, hirsutism and blood sample data obtained from this study Click here for additional data file. Copyright: © 2019 Hestiantoro A et al. 2019 Data associated with the article are available under the terms of the Creative Commons Zero "No rights reserved" data waiver (CC0 1.0 Public domain dedication).

## Discussion

This study was conducted to determine factors that influence the hyperandrogenism phenotype in PCOS patients, more specifically the concordance between its clinical features and biochemical parameters. Even though hyperandrogenism is one of the pivotal features of PCOS, not every patient with PCOS exhibits such hyperandrogenic phenotype. Pathophysiologically, hyperandrogenism is associated with intense ovarian steroidogenesis due to thecal cell hyperplasia. There are marked increases in GnRH and LH secretion, along with relative deficit in FSH secretion, which resulted in aberrant follicle growth and development, as well as reduced conversion of androstenedione and dehydroepiandrosterone to estrogen ^3, 11^. Insulin resistance also plays a significant role in the development of hyperandrogenism, by increasing the secretion pulse of LH and suppressing the production of SHBG in the liver, thus increasing the level of testosterone ^3^.

This study found that there were three biochemical parameters that significantly differed between the two groups and correlated with hyperandrogenism phenotype, which were elevated testosterone and triglyceride levels, as well as decreased SHBG level. Testosterone and triglyceride levels had positive correlations with FTI, while SHBG showed negative correlation with FTI. There is an inverse relationship between SHBG and free testosterone level. SHBG is a glycoprotein that binds and transports sex steroids, such as testosterone and estradiol in the plasma. SHBG concentration is strongly influenced by various factors, such as sex steroid balance, drugs, thyroid hormone, insulin, dietary composition, and liver diseases. Lower level of SHBG means that less testosterone is bound, which results in a higher free testosterone concentration detected in blood plasma ^12, 13^.

We also found triglyceride levels to be a determinant factor of hyperandrogenism, with a weak positive correlation with FTI. Dyslipidemia is one of the most commonly found metabolic disturbances in PCOS, occurring in approximately 70% of PCOS patients. The pathogenic mechanisms underlying this condition are complex and are not yet fully understood. Previous studies found that obesity, hyperandrogenism, and hyperinsulinemic insulin resistance contributed to the development of hypertriglyceridemia in PCOS. Hyperandrogenism and hyperinsulinemia cause defective cathecolamine-induced lipolysis which eventually leads to the increased release of free fatty acids. Free fatty acids then stimulate hepatic overproduction of VLDL with more triglyceride compounds on each VLDL particles. Hyperandrogenism is also believed to have crucial roles in the upregulation of several genes which involved in the catabolism of lipoproteins, such as scavenger receptor B1 (SR-B1) and hepatic lipase (HL). Therefore, it is foreseeable that hyperandrogenic patients will have significantly higher level of triglyceridemia, compared to those with normal androgen concentration ^14, 15^.

Even though not explicitly stated in this study, an independent relationship was observed between triglyceride level and insulin resistance. A moderate positive correlation was observed between HOMA-IR and triglyceride level in patients with PCOS (p < 0.001, r = 0.445). Triglyceride levels are considered a useful marker in identifying insulin resistance, particularly in patients with metabolic syndrome ^16^. As stated above, insulin resistance, along with its compensatory hyperinsulinemia, contributed to triglyceride dysregulation in hyperandrogenic patients with PCOS. Hyperinsulinemia inhibits microsomal triglyceride protein expression which is crucial in the regulation of apolipoprotein B-100 and VLDL production. It also suppresses the removal of triglyceride-rich protein. Insulin resistant PCOS patients are more prone to dysregulation of lipid metabolism compared to those with normal insulin sensitivity (81% vs. 65%, respectively) ^14, 15^. A recently published study revealed a two-way relationship between androgen excess and insulin resistance. FAI as the indicator of hyperandrogenism can serve as an indicator of glucose tolerance, as an increase in FAI is usually followed by increases in blood glucose concentration, insulin level, and glucose resistance ^17^.

A novel concept, dysbiosis of gut microbiota (DOGMA), has been found to have considerable impact on the pathogenesis of PCOS, particularly through the development of insulin resistance and hypertriglyceridemia. A high-fat/high-sugar diet and obesity are the primary causes of DOGMA, driving increases in the growth of pathogenic microorganisms and suppress the growth good bacteria, which further leads to metabolic endotoxemia (the leakage of lipopolysaccharides produced by Gram-negative bacteria to systemic circulation) and chronic low-grade inflammatory conditions in the gut. Chronic low-grade inflammation interferes with islet *β*-cell proliferation and insulin receptor function, thus resulting in insulin resistance and compensatory hyperinsulinemia. In addition to that, DOGMA also plays a role in the development of dyslipidemia by modulating hepatic and systemic metabolism of lipid and glucose via the elicitation of short-chain fatty acids ^18– 22^.

Aside from DOGMA, vitamin D deficiency has also been implicated in insulin resistance and dyslipidemic condition commonly found in patients with PCOS. Many previous studies have indicated that PCOS patients with vitamin D deficiency tend to have higher levels of triglyceride and Homa-IR, compared to those with sufficient level of vitamin D concentration. Physiologically speaking, the vitamin D–vitamin D receptor (VDR) complex enacts an important role in regulating several genes, including those involved in glucose and lipid metabolism. Therefore, it is likely that interference in vitamin D concentration would also disrupt the metabolism of glucose and lipid ^23– 25^. One interesting finding to be noted in this study is the fact that no statistically significant difference was found between FGS and FTI. This finding implicates that the symptoms of hyperandrogenism in our PCOS subjects could not be assessed using FGS. The reason underlying this finding is the fact that clinical signs of hyperandrogenism are not particularly noticeable in PCOS patients in Asian countries, including Indonesia. Hirsutism appearance on each individual depends on their sensitivity to circulating androgens and its variation is influenced by ethnicity. Asian women tend to be less hirsute than Caucasian women, despite the elevated levels of androgen ^26^. A number of controversies regarding the extent of testosterone level and FTI in predicting the severity of hirsutism have prevailed upon earlier studies ^6, 27^. Pathophysiologically, androgens play important role in the growth of terminal hair on several predilection areas which are normally hairy for men, but not for women ^7^. However, prior clinical study discovered that only 68% hirsute PCOS patients were hyperandrogenic and only 63% hyperandrogenic PCOS patients were diagnosed with hirsutim ^7, 28^. This implicates that there are other factors that might contribute to hyperandrogenism other than hirsutism, vice versa.

## Conclusions

In conclusion, a high FTI in PCOS patients is associated with high triglyceride levels, high testosterone levels, and low SHBG levels. Ferriman Gallwey score, as an indicator of hirsutism, shows no significant association with FTI. These associations mean that hyperandrogenic phenotype of PCOS patients is influenced by both hormonal and metabolic dysfunctions.

## Data availability

The data referenced by this article are under copyright with the following copyright statement: Copyright: © 2019 Hestiantoro A et al.

Data associated with the article are available under the terms of the Creative Commons Zero "No rights reserved" data waiver (CC0 1.0 Public domain dedication). 

Dataset 1. Anthropometric, hirsutism and blood sample data obtained from this study. DOI: https://doi.org/10.5256/f1000research.16815.d231733 ^29^.
