# Supplementary material for: Triglycerides, independent of Ferriman Gallwey Score, is a main determinant of free testosterone index in PCOS
Source: F1000Res. 2019 Jan 23;8:94. [Version 1] doi: 10.12688/f1000research.16815.1 (PMC6406175; doi:10.12688/f1000research.16815.1)
Supplement: Peer review history

# Peer review: Reviewer response for version 1

**DOI:** 10.5256/f1000research.18382.r44622

**Published:** 2019-03-07

**Authors:** Zheying Min

**Affiliations:** 1 Beijing Key Laboratory of Reproductive Endocrinology and Assisted Reproductive Technology, Key Laboratory of Assisted Reproduction, Ministry of Education, Department of Obstetrics and Gynecology, Center for Reproductive Medicine, Peking University Third Hospital, Beijing, China; 2 Center for Life Sciences, Academy for Advanced Interdisciplinary Studies, Peking University, Beijing, China

This study describes the correlation between hormone profile, lipid profile and clinical profile with free testosterone index in subjects with PCOS. Triglycerides, testosterone, and SHBG were associated with hyperandrogenism in PCOS patients, while FGS showed no such association.

 Overall the paper is well structured and written, with results and a strong discussion. However I would have the following comments to the paper:

1. The average BMI of all PCOS patients were more than 25 (27.6). Could obesity influence the statistical analysis of the hormone?
2. There are some evident errors in Tables. For example, In Table 1, is the average value of the body weight 0.6?
3. The manuscript must be edited again for typo errors. In the discussion, “There are marked increases in GnRH and LH secretion, along with relative deficit in FSH secretion……” I think it should change deficit to deficiency.
4. There are too many references in the discussion part, which should be moved to the introduction part. Because it only includes 9 references in the introduction.

I have read this submission. I believe that I have an appropriate level of expertise to confirm that it is of an acceptable scientific standard.

# Peer review: Reviewer response for version 1

**DOI:** 10.5256/f1000research.18382.r43535

**Published:** 2019-02-05

**Authors:** Xue Lian Li, Dan-Feng Du

**Affiliations:** 1 Department of Gynaecology, OB/GYN Hospital, Fudan University, Shanghai, China; 2 Shanghai Key Laboratory of Female Reproductive Endocrine-Related Diseases, Shanghai, China

Polycystic Ovary Syndrome (PCOS) is characterized by a series of endocrine and metabolism disturbances, such as insulin resistance, hyperandrogenism, sympathetic dysfunction and chronic low-grade inflammation state ^1^ but the inter-relationships between these factors still remain unclear. Hyperandrogenism is the most important manifestation and diagnostic criteria of PCOS, and the Androgen Excess and PCOS Society (AES) has proposed that hyperandrogenism should be the essential condition to diagnose PCOS ^2^.There are three forms of serum testosterone (T), 60-65% was combined to sex hormones lobulin (SHBG) tightly, 35-40% was combined to albumin and free testosterone (FT) only consists 1-2% of total T. Which kind of serum androgen should be measured for diagnosis of PCOS remains controversial. Recently, it is believed assessments of free testosterone levels are more sensitive than the measurement of total T for establishing the existence of androgen excess ^3^. Dr. Hestiantoro aims to determine the correlation between hormone profile, lipid profile and clinical profile with free testosterone index in subjects with PCOS, which is worthy of study.

 According to the Rotterdam diagnostic criteria, there are at least four phenotypes of PCOS: Subtype I - PCO & hyperandrogenism & oligo-ovulation, SubtypeⅡ - PCO & oligo-ovulation, SubtypeⅢ - hyperandrogenism & oligo-ovulation, Subtype Ⅳ - PCO + hyperandrogenism. Different phenotypes may display different endocrine disorders.

 Dr. Hestiantoro has shown that high FTI in PCOS patients is associated with high triglyceride levels, high testosterone levels, and low SHBG levels, while Ferriman Gallwey score, as an indicator of hirsutism, shows no significant association with FTI. But another researcher who assessed the lipid profile in lean and non-lean PCOS patients, hyperandrogenemia was defined as free androgen index (FAI) ≥5, whose results show higher levels of total cholesterol, high-density lipoprotein cholesterol in lean patients with FAI <5 than in lean patients with FAI ≥ 5. There were no differences in lipid profile between non-lean patients with FAI ≥ 5 and non-lean patients with FAI <5 ^4^. Another study has also confirmed these results with Ferriman-Gallwey scores(FGS) and triglycerides are significantly higher in PCOS patients ^5^. In another study, PCOS patients with adrenal hyperandrogenism do not exhibit deterioration in insulin resistance and lipid profile despite the higher degree of total androgens ^6^. 

 So what is the real correlation between hormone profile, lipid profile, and clinical profile with free testosterone index in subjects with PCOS? In my opinion, different PCOS phenotypes may display different endocrine and metabolic disorders, and FTI (free testosterone index) is a very valuable potential measurement to diagnose PCOS. It is of great significance to identify endocrine and metabolic characteristics of different phenotypes, but I am afraid the sample size of Dr.Hestiantoro's research is still too small to answer this question, and I suggest the authors may clarify PCOS patients to more detailed phenotypes and may have more interesting findings with a bigger sample size.

We have read this submission. We believe that we have an appropriate level of expertise to confirm that it is of an acceptable scientific standard.

## References

[1] ShorakaeSRanasinhaSAbellSLambertGLambertEde CourtenBTeedeH: Inter-related effects of insulin resistance, hyperandrogenism, sympathetic dysfunction and chronic inflammation in PCOS. *Clin Endocrinol (Oxf)*.2018;89(5) : 10.1111/cen.13808 628-633 10.1111/cen.13808 29992612

[2] Thessaloniki ESHRE/ASRM-Sponsored PCOS Consensus Workshop Group: Consensus on infertility treatment related to polycystic ovary syndrome. *Hum Reprod*.2008;23(3) : 10.1093/humrep/dem426 462-77 10.1093/humrep/dem426 18308833

[3] GoodmanNFCobinRHFutterweitWGlueckJSLegroRSCarminaEAmerican Association of Clinical Endocrinologists AACEAmerican College of Endocrinology ACEAndrogen Excess and PCOS Society AES: AMERICAN ASSOCIATION OF CLINICAL ENDOCRINOLOGISTS, AMERICAN COLLEGE OF ENDOCRINOLOGY, AND ANDROGEN EXCESS AND PCOS SOCIETY DISEASE STATE CLINICAL REVIEW: GUIDE TO THE BEST PRACTICES IN THE EVALUATION AND TREATMENT OF POLYCYSTIC OVARY SYNDROME--PART 1. *Endocr Pract*.2015;21(11) : 10.4158/EP15748.DSC 1291-300 10.4158/EP15748.DSC 26509855

[4] SpałkowskaMMrozińskaSGałuszka-BednarczykAGosztyłaKPrzywaraAGuzikJJaneczkoMMilewiczTWojas-PelcA: The PCOS Patients differ in Lipid Profile According to their Phenotypes. *Exp Clin Endocrinol Diabetes*.2018;126(7) : 10.1055/s-0043-121264 437-444 10.1055/s-0043-121264 29388179

[5] InalZOErdemSGederetYDuranCKucukaydinZKurkuHSakaryaDK: The impact of serum adropin and ischemia modified albumin levels based on BMI in PCOS. *Endokrynol Pol*.2018;69(2) : 10.5603/EP.a2018.0002 135-141 10.5603/EP.a2018.0002 29465156

[6] PaschouSAPaliouraEIoannidisDAnagnostisPPanagiotakouALoiVKarageorgosGGoulisDGVryonidouA: Adrenal hyperandrogenism does not deteriorate insulin resistance and lipid profile in women with PCOS. *Endocr Connect*.2017;6(8) : 10.1530/EC-17-0239 601-606 10.1530/EC-17-0239 28912337PMC5640571
